# Ursolic Acid Simultaneously Targets Multiple Signaling Pathways to Suppress Proliferation and Induce Apoptosis in Colon Cancer Cells

**DOI:** 10.1371/journal.pone.0063872

**Published:** 2013-05-30

**Authors:** Jingshu Wang, Liqun Liu, Huijuan Qiu, Xiaohong Zhang, Wei Guo, Wangbing Chen, Yun Tian, Lingyi Fu, Dingbo Shi, Jianding Cheng, Wenlin Huang, Wuguo Deng

**Affiliations:** 1 State Key Laboratory of Oncology in South China, Sun Yat-Sen University Cancer Center, Guangzhou, China; 2 Institute of Cancer Stem Cell, Dalian Medical University Cancer Center, Dalian, China; 3 The First Affiliated Hospital-Huangpu Hospital, Sun Yat-Sen University, Guangzhou, China; 4 Department of Forensic Pathology, Sun Yat-Sen University Medical School, Guangzhou, China; 5 State Key Laboratory of Targeted Drug for Tumors of Guangdong Province, Guangzhou Double Bioproduct Inc, Guangzhou, China; Albany Medical College, United States of America

## Abstract

Ursolic acid (UA), a natural pentacyclic triterpenoid carboxylic acid distributed in medical herbs, exerts antitumor effects and is emerging as a promising compound for cancer prevention and therapy, but its excise mechanisms of action in colon cancer cells remains largely unknown. Here, we identified the molecular mechanisms by which UA inhibited cell proliferation and induced apoptosis in human colon cancer SW480 and LoVo cells. Treatment with UA led to significant inhibitions in cell viability and clone formation and changes in cell morphology and spreading. UA also suppressed colon cancer cell migration by inhibiting MMP9 and upregulating CDH1 expression. Further studies showed that UA inhibited the phosphorylation of Akt and ERK proteins. Pretreatment with an Akt or ERK-specific inhibitor considerably abrogated the proliferation inhibition by UA. UA also significantly inhibited colon cancer cell COX-2 expression and PGE2 production. Pretreatment with a COX-2 inhibitor (celecoxib) abrogated the UA-induced cell proliferation. Moreover, we found that UA effectively promoted NF-κB and p300 translocation from cell nuclei to cytoplasm, and attenuated the p300-mediated acetylation of NF-κB and CREB2. Pretreatment with a p300 inhibitor (roscovitine) abrogated the UA-induced cell proliferation, which is reversed by p300 overexpression. Furthermore, UA treatment induced colon cancer cell apoptosis, increased the cleavage of PARP, caspase-3 and 9, and trigged the release of cytochrome c from mitochondrial inter-membrane space into cytosol. These results indicate that UA inhibits cell proliferation and induces apoptosis in colon cancer cells through simultaneous modulation of the multiple signaling pathways such as MMP9/CDH1, Akt/ERK, COX-2/PGE2, p300/NF-κB/CREB2, and cytochrome c/caspase pathways.

## Introduction

Colon and rectal cancer (colorectal cancer, CRC), the third most common cancer worldwide, has become one of the leading causes of death from cancers [1]. Surgery, chemotherapy and radiotherapy are the primary and common treatments for colorectal cancer. Although chemotherapy is adjuvant to surgery, the cure rate of colorectal cancer was still not ideal, especially for the later stage patients. The metastasis and recurrence after surgery or the produced chemotherapy resistance usually leads to the final death. Thus, complementary and alternative treatment strategy has become necessary to improve the survival rate of colon cancer patients. Chinese herbal medicine is becoming more and more popular in cancer treatments combined with conventional therapy due to its natural origin, low toxicity and effectiveness to prevent and treat cancers, including colon cancer [2].

Ursolic acid (UA), a natural pentacyclic triterpenoid carboxylic acid extracted from medical herbs and edible plants, exerts a wide range of biological activities, including hepatoprotective [3], anti-bacterial [4], antiviral [5], and anti-inflammatory [6]. Furthermore, it has been implicated in prevention and protection against cancers [7]. Its anti-tumor action is attributed to its ability to prevent tumorigenesis [8], inhibit cancer cell proliferation, and induce cancer cell apoptosis [6], [9]. The signaling pathways involved in UA activity might be different in different cancer cell lines, and partial signaling pathways might be regulated simultaneously or successively and synergized to contribute to UA treatment. However, the precise molecular mechanisms of UA involved in proliferation inhibition and apoptosis induction in colorectal cancer were still not clear enough.

The enzyme cyclooxygenase-2 (COX-2), responsible for the catalysis of the conversion of arachidonic acid to prostaglandins and thromboxane A_2_, is known to be involved in multiple pathophysiological processes, including inﬂammation and tumorigenesis [10], [11]. COX-2 is undetectable in most normal tissues, but it is commonly overexpressed in many premalignant, malignant and metastatic human cancers, including colorectal cancer, with its downstream product prostaglandin E2 (PGE2). The increasing evidence indicated the key roles of COX-2 in carcinogenesis and cancer progression are through participating in tumor initiation, promoting tumor maintenance and progression, and encouraging tumor metastatic spread [12], [13]. The selective inhibition of COX-2 activity reverses carcinogenesis of colorectal cancer and has been shown to induce apoptosis, and inhibit proliferation and angiogenesis [14], [15]. Some of its inhibitors even have been shown to be potentially attractive chemotherapeutic drugs in the treatment of colorectal cancer combined with other common chemotherapeutic agents [16], [17]. COX-2 expression is tightly regulated at the transcription level through the binding of transactivators such as NF-κB, CREB2 and co-activators such as p300 to the corresponding sites located in its promoter [18]–[21]. However, whether UA plays its anti-tumor effect through regulations of the COX-2, NF-κB and p300 signaling is still poorly understood in human colorectal cancer, and the related underlying signaling pathways remain elusive.

Here, we evaluated the effect of UA on colon cancer cell proliferation, migration, and apoptosis in colon cancer cells and analyzed its regulation on the key proteins of some signaling pathways involved in cell proliferation, migration and apoptosis. The results showed the anti-proliferation, anti-migration and pro-apoptotic effects of UA in colon cancer cells were mediated through simultaneous modulation of multiple signaling pathways, including MMP9/CDH1, Akt/ERK, COX-2/PGE2, p300/NF-κB/CREB2 and cytochrome c/caspase-dependent pathways. Our study not only uncovers the underlying molecular mechanisms of UA action in human colon cancer cells, but also suggests the great potential of UA in human colorectal cancer prevention and treatment.

## Results

### UA Inhibited Cell Proliferation and Clone Formation and Induced Cell Morphology Change

**Figure 1:**
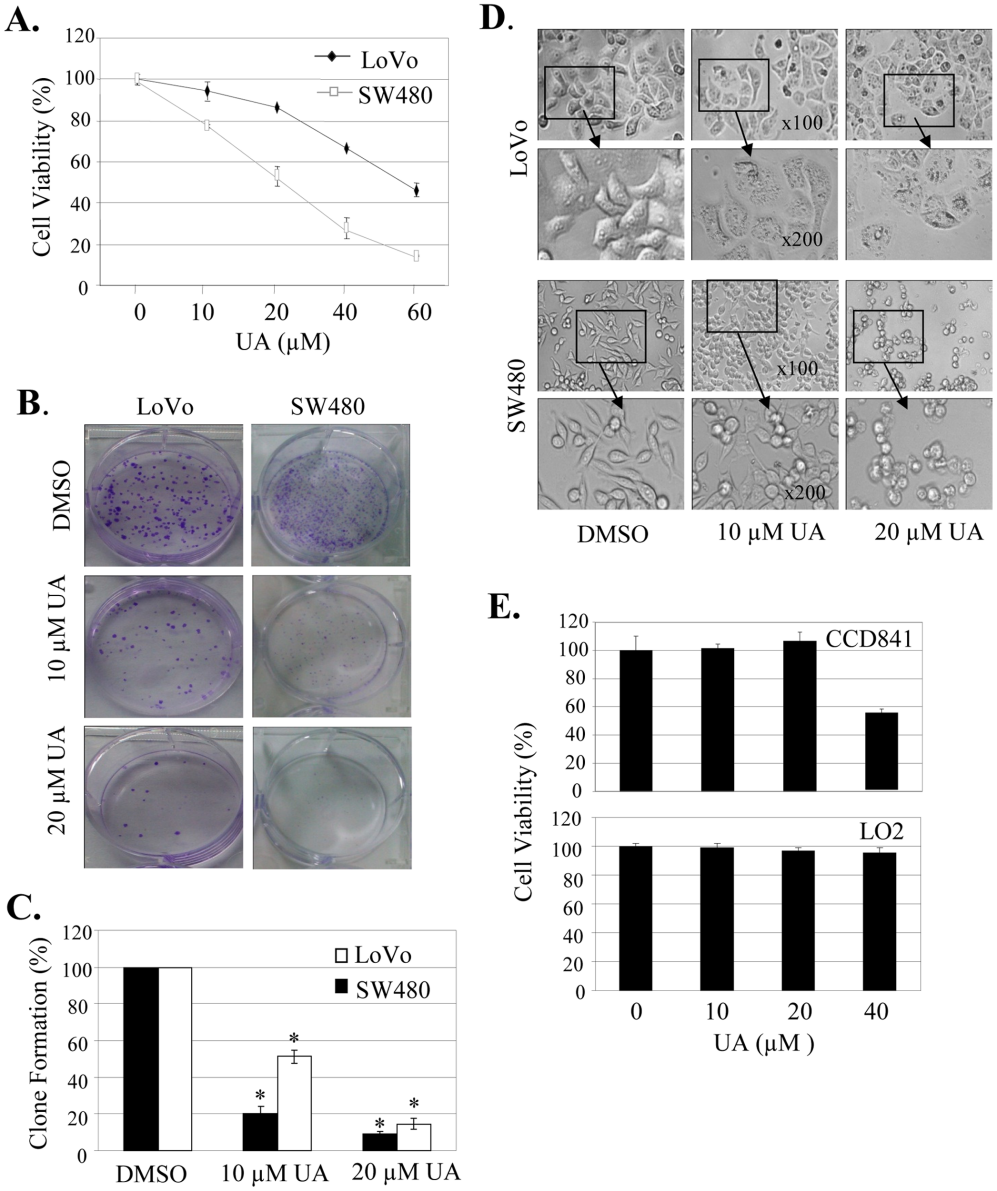
UA induced cell proliferation inhibition and morphology change. Human colon cancer cell line SW480 and LoVo and normal cell lines CCD841 and LO2 were treated with UA at the indicated doses. At 48 hours after treatment, cell viability was determined by a MTT assay (**A, E**)**,** and the tumor cell-induced clone formation was analyzed (**B, C**). The changes in cell morphology, spreading and blebbing in cells treated without or with UA (10 µM and 20 µM) for 48 h were observed and cells were photographed using a microscope fitted with digital camera (**D**). The percent cell viability or clone formation ratio in each treatment group was calculated relative to cells treated with DMSO vehicle control. The data are presented as mean ± SD of three separate experiments. *, *P*<0.05, significant differences between treatment groups and DMSO control groups.

We first quantitatively analyzed the effect of UA on cell proliferation at 48 hours after treatment in SW480 and LoVo cells by a MTT assay. Treatment with UA at the dose of 10 µM to 60 µM significantly inhibited cell viability in a concentration-dependent manner, resulting in a 20% to an 83% inhibition in SW480 cells and a 5% to 55% inhibition in LoVo cells, respectively (Fig. 1A). We also determined the effects of UA on tumor cell clonogenicity. UA also markedly inhibited clone formation in a dose-dependent manner (Fig. 1B), resulting in a significant inhibition of clone formation ratio in both SW480 and LoVo cells (Fig. 1C).

We next analyzed the UA-mediated changes in cell morphology and spreading in SW480 and LoVo cells. Treatment of cells with DMSO, a vehicle control, formed a cell layer, and more spreading, filopodia and blebbing were observed. By contrast,UA treatment caused a marked reduction of cell-to-cell contact, and had lower spreading with fewer formation of filopodia in both SW480 and LoVo cells, and induced membrane blebbing in cell treated with UA (Fig. 1D).

We also evaluated the effect of UA on cell proliferation in human normal cell lines CCD841 and LO2. Treatment with UA at the doses ≤20 µM did not inhibit cell viability in both normal cell lines, demonstrating the potential specificity of UA in inhibiting tumor cell proliferation at the dose ≤20 µM.

### UA Targeted MMP9/CDH1 Signaling to Inhibit Cell Migration

**Figure 2:**
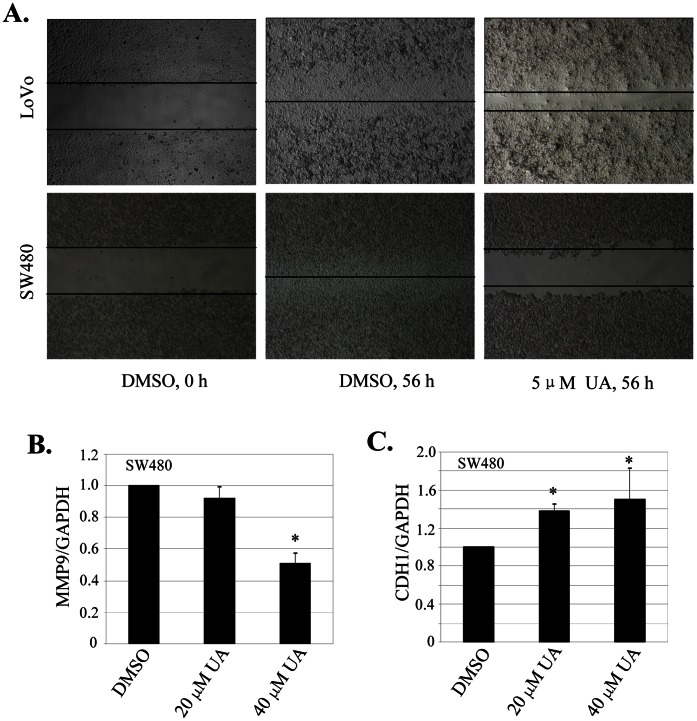
UA inhibited cell migration inhibition by regulating MMP9 and CDH1 expression. (**A**), Cell migration was analyzed by a scratch assay. SW480 and LoVo cells were grown to full confluency. The cell monolayers were wounded with a sterile pipette tip, and washed with medium to remove detached cells from the plates. Cells were left either untreated or treated with indicated doses of UA. After 56 h, the wound gap was observed and cells were photographed. (**B, C**)**,** SW480 cells were treated with UA at the indicated doses. At 56 hours after treatment, the expression of MMP9 (**B**) or CDH1 (**C**) was quantitatively analyzed by a real-time qPCR analysis. *, *P*<0.05, significant differences between the UA-treated groups and the DMSO-treated groups.

We next examined the effect of UA on cell migration by employing a scratch assay. Consistent with the data from cell proliferation and clonogenicity inhibition, treatment with UA at 5 µM effectively inhibited cell migration in both SW480 and LoVo cells (Fig. 2A). The part of gap or wounding space between cell layers after making a scratch was occupied completely by the migrating cells after 56 h. By contrast, the empty space of the cells was not occupied by the migrating cells treated with UA (Fig. 2A).

MMP9 and CDH1 are two key molecules involved in the cell invasion and migration-related signaling pathway [22], [23]. We next quantitatively analyzed the effects of UA on the expression of MMP9 and CDH1 by a real-time qPCR analysis in SW480 cells. As expected, UA at 20 µM and 40 µM markedly suppressed the mRNA level of MMP9 gene (Fig. 2B), whereas UA treatment led to a significant increase in CDH1 mRNA expression (Fig. 2C). These results indicate that UA plays an important role in suppressing migration and invasion via modulation of MMP9 and CDH1 signaling.

### UA Inactivated Akt and ERK Signaling to Inhibit Cell Proliferation

**Figure 3:**
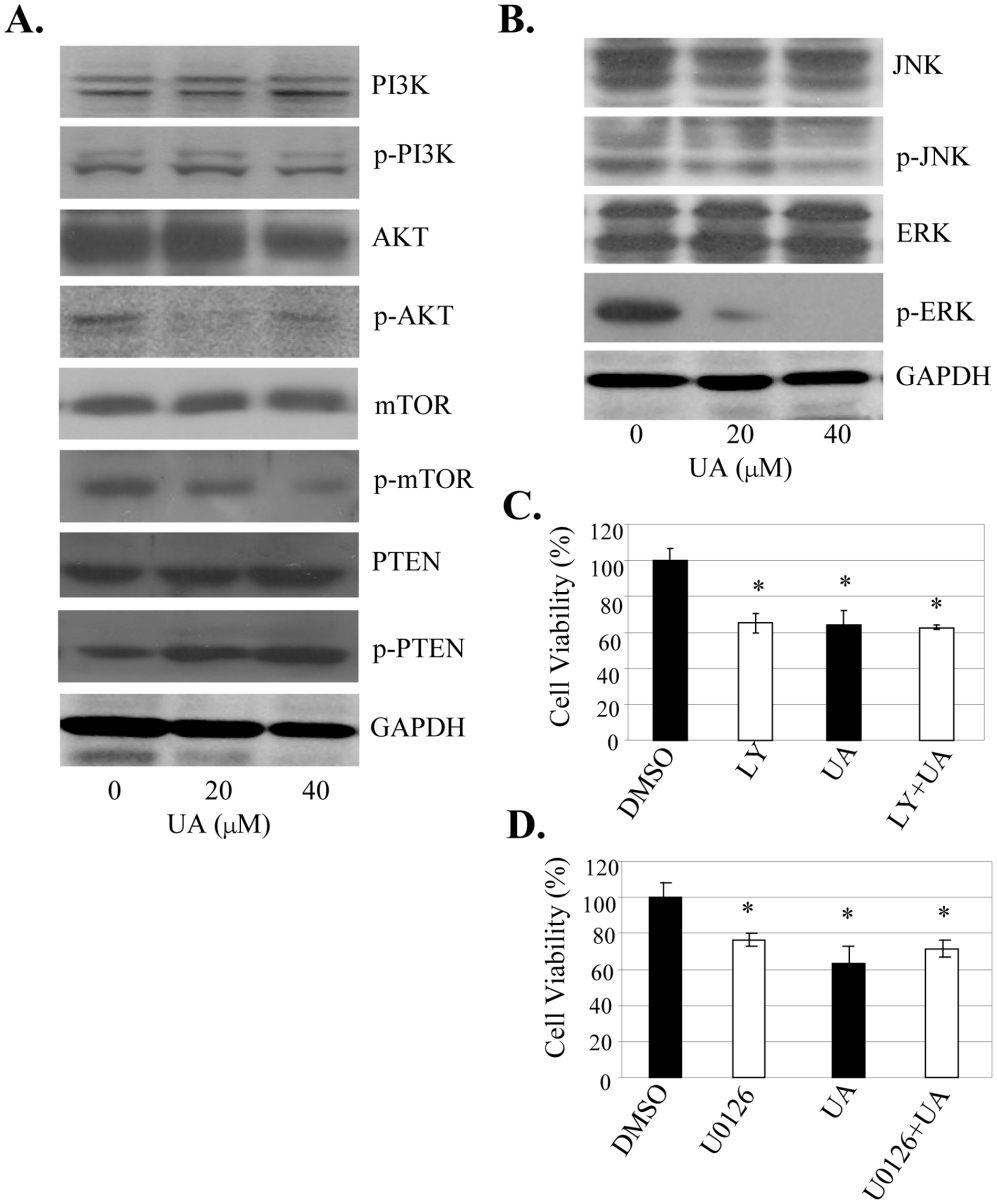
UA inactivated Akt/ERK signaling to inhibit cell proloferation. (**A, B**)**,** SW480 cells were treated with UA at 20µM or 40 µM for 48 h. The expression of the phosphorylated or total PI3K, Akt, mTOR, PTEN, JNK, ERK1/2 proteins was detected by Western blot. GAPDH was used as a control for sample loading. (**C, D**)**,** SW480 cells were treated with a PI3K/Akt-selective inhibitor LY294002 (LY, 5 µM) (**C**) or with an ERK-selective inhibitor U0126 (20 µM) (**D**) for 4 hours, and then treated with UA at 20 µM. At 48 hours after treatment, cell viability was determined by MTT analysis. The percent cell viability in each treatment group was calculated relative to cells treated with the vehicle control DMSO. The data are presented as the mean ± SD of three separate experiments. *, *P*<0.05, significant differences between treatment groups and control groups.

The PI3K/Akt and MAPK signaling pathways play critical roles in controlling cell proliferation and tumorigenesis. To evaluate whether UA is capable of modulating the PI3K/Akt and MAPK signaling to lead to inhibition of cell proliferation, we examined the effects of UA on phosphorylation of the signaling molecules in these two pathways in SW480 cells by Western blot analysis. Treatment with UA at 20 µM considerably inhibited the expression of phosphorylated Akt and mTOR protein, but increased phosphorylated PTEN expression (Fig. 3A). We also detected a marked reduction in the phosphorylated ERK proteins in a dose-dependent manner (Fig. 3B). The levels of the total protein expression of Akt, mTOR, PTEN (Fig. 3A) and ERK (Fig. 3B) were not changed.

To further confirm the involvement of the Akt and ERK signaling pathways in the UA-mediated inhibition of cell proliferation, we next analyzed the effects of the Akt or ERK-selective inhibitor on UA-mediated proliferation inhibition in SW480 cells. Pretreatment of cells with PI3K/Akt inhibitor LY294002 (LY) at 5µM (Fig. 3C) or ERK Inhibitor U0126 at 20 µM (Fig. 3D) inhibited cell proliferation, and a combination of UA (20 µM) with these inhibitors slightly increased the inhibition of cell proliferation (Fig. 3C and 3D). However, addition of UA did not significantly change the proliferation inhibition in the SW480 cells pretreated with Akt inhibitor LY (Fig. 3C) or ERK inhibitor U0126 (Fig. 3D), confirming the role of UA in regulating the Akt and ERK signaling.

### UA Targeted COX-2 and PGE2 Signaling to Inhibit Cell Proliferation

**Figure 4:**
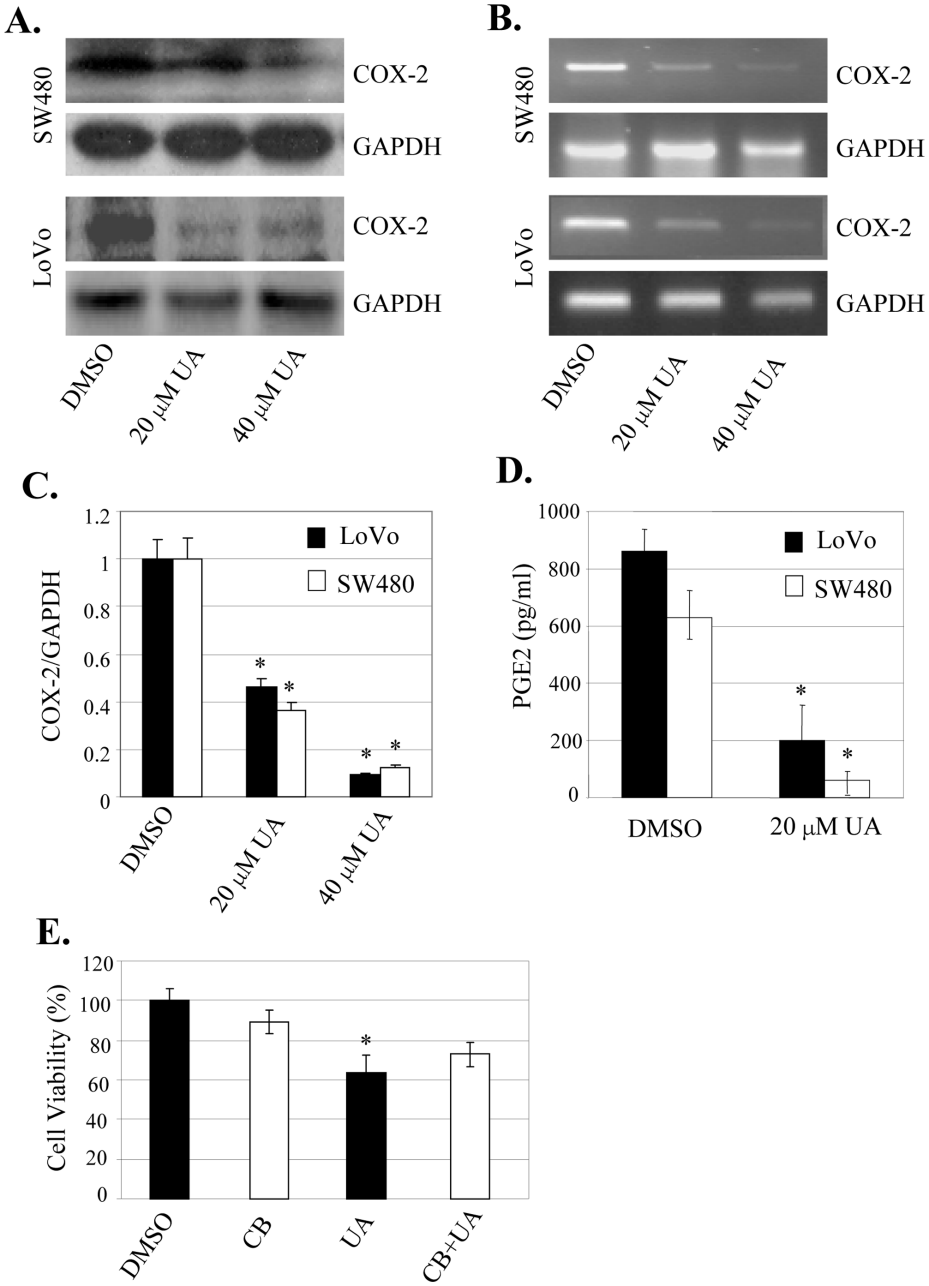
UA suppressed COX-2/PGE2 signaling to inhibit cell proliferation. (A–D), SW480 and LoVo cells were treated with UA for 48 h. The expression of COX-2 protein (**A**) and mRNA (**B**) were analyzed by Western blotting and RT-PCR, respectively. The densities of COX-2 mRNA and the ratio of COX-2/GAPDH were quantitatively analyzed (**C**). The production of PGE2 was detected by ELSIA analysis (**D**). GAPDH was used as controls for sample loading. (**E**)**,** SW480 cells were treated with a COX-2-selective inhibitor celecoxib (CB, 20 µM) for 4 hours, and then treated with UA at 20 µM. At 48 hours after treatment, cell viability was determined by MTT analysis. The data are presented as the mean ± SD of three separate experiments. *, *P*<0.05, significant differences between treatment groups and control groups.

COX-2 expression has been shown to upregulate the EGFR, PI3K/Akt and ERK signaling, thereby induce tumor cell proliferation, migration and invasion [24], [25]. To determine whether the UA-mediated inhibition of cell proliferation is mediated through modulation of the COX-2 signaling in colon cancer cells, we evaluated the effect of UA on COX-2 expression at protein and mRNA levels by Western blot and RT-PCR. Treatment with UA at the doses of 20 µM and 40 µM dramatically inhibited the expression of COX-2 protein (Fig. 4A) and mRNA (Fig. 4B) in SW480 and LoVo cells. The quantitative densitometry analysis also showed that UA at 20 µM and 40 µM significantly suppressed COX-2 mRNA in both SW480 and LoVo cells (Fig. 4C).

PGE2 is a downstream product of COX-2, and it is synthesized via the cyclooxygenase and prostaglandin synthase pathways. We treated SW480 and LoVo cells with UA at 20 µM and determined the effect of UA on PGE2 production. The results showed that UA treatment significantly reduced PGE2 production in both cells (Fig. 4D).

To further confirm that the UA-mediated inhibition of cell proliferation is through regulation of COX-2 signaling in colon cancer cells, we pretreated SW480 cells with celecoxib (CB, 10 µM), a COX-2-selective inhibitor, and tested the effect of UA on the celecoxib-mediated inhibition of cell proliferation. As shown in Fig. 4E, pretreatment with celecoxib (CB) considerably inhibited cell viability, whereas UA at 20 µM did not significantly alter the inhibition mediated by celecoxib. These results showed that the inhibition of colon cancer cell proliferation by UA might be also partially mediated by inhibiting the COX-2 signaling.

### UA Induced Translocation of NF-κB and p300 from Cell Nuclei to Cytoplasm

**Figure 5:**
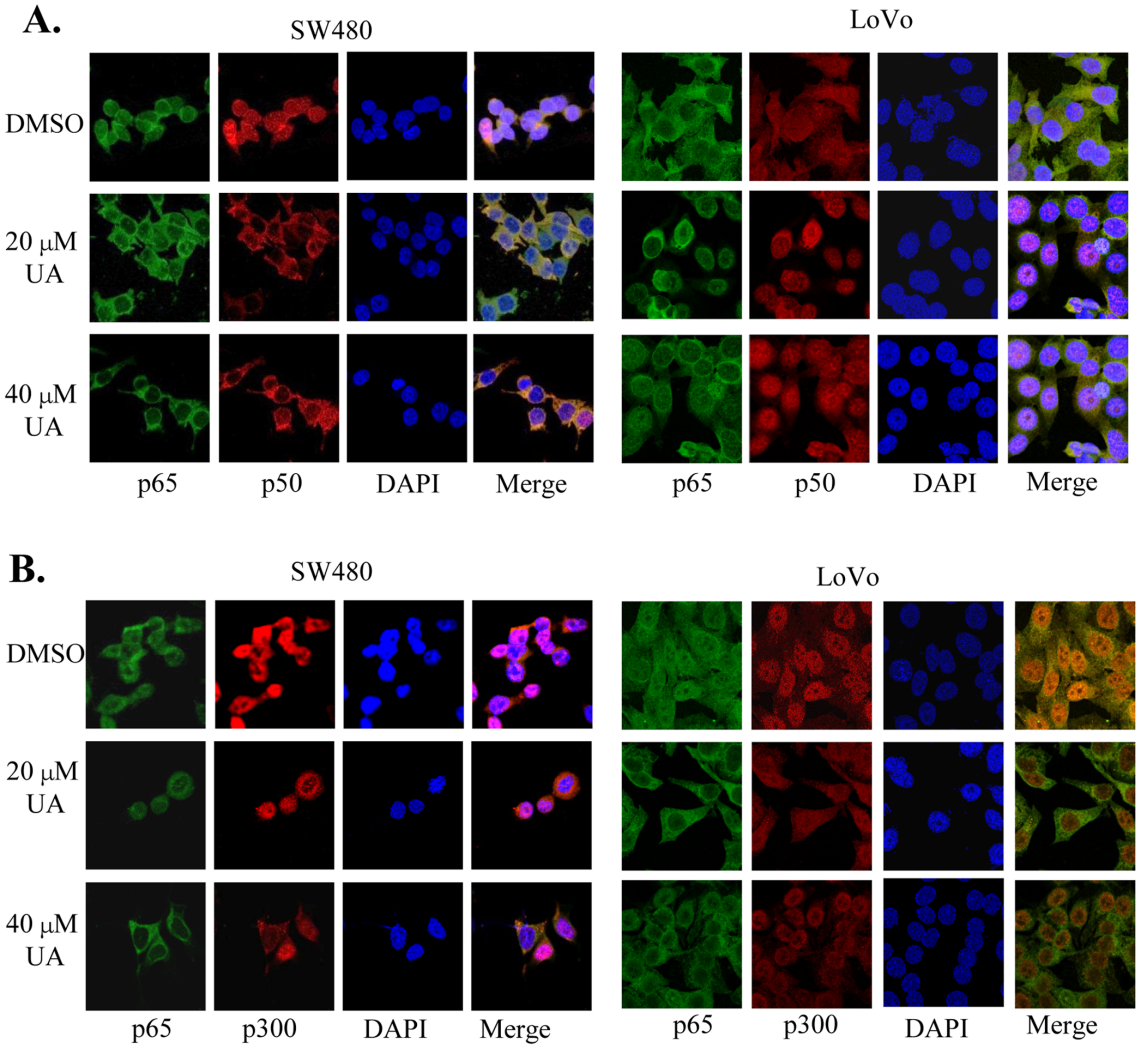
UA induced the translocation of NF-κB and p300 from nuclei to cytoplasm. Human SW480 and LoVo cells grown on chamber slides were treated with UA. At 48 hours after treatment, the sub-cellular localization of p50, p65 and p300 and the co-localization of p65 with p50 (**A**) or p300 (**B**) were examined by confocal microscopy analysis with a confocal microscope. More than 100 cells were inspected per experiment, and cells with typical morphology were presented.

The expression of COX-2 is regulated by the translocation and interaction of transactivator NF-κB and coactivator p300 in tumor cell nuclei. We next performed immunofluorescence assay to evaluate the effect of UA on nuclear localization and interaction of the NF-κB and p300 in colon cancer SW480 and LoVo cells. We detected the constitutive translocation of p50 and p65 NF-κB and p300 to cell nuclei (Fig. 5A and 5B) and the co-localization of p65 with p50 (Fig. 5A) or p300 (Fig. 5B) in both SW480 and LoVo cells. Treatment with UA at 20 µM and 40 µM induced translocation of the NF-κB (Fig. 5A) and p300 (Fig. 5B) from cell nuclei to cytoplasm. The results indicate that UA targeted the NF-κB and p300 signaling by promoting their translocation from cell nuclei to cytoplasm in colon cancer cells.

### UA Targeted p300 Signaling to Inhibit NF-κB/CREB-2 Acetylation and Cell Proliferation

**Figure 6:**
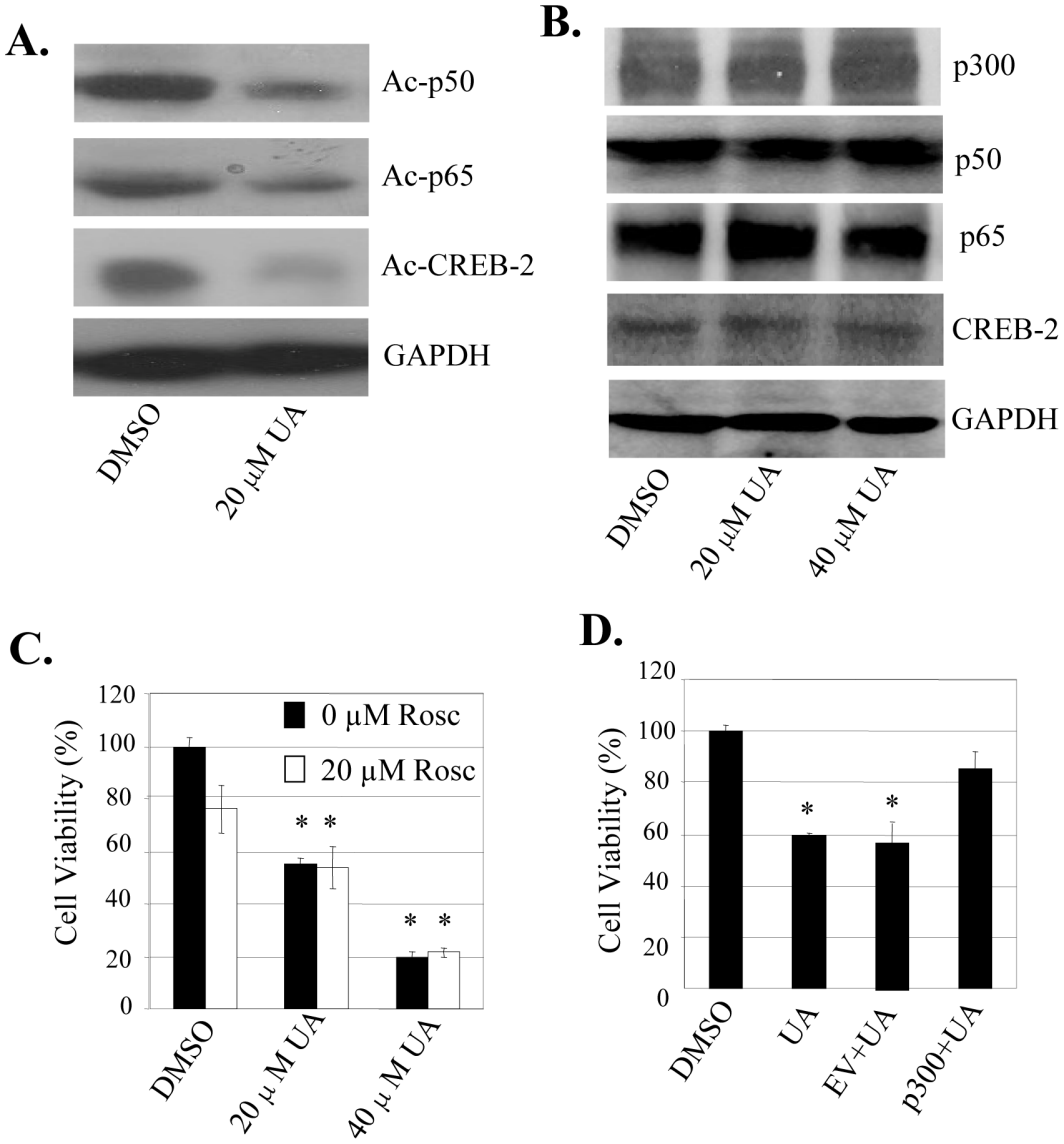
UA targeted p300 signaling to inhibit NF-κB and CREB2 acetylation and cell proliferation. (**A, B**)**,** SW480 cells were transfected with FLAG-p300 for 24 h and then treated with UA for 48 h. The nuclear extracts were prepared and p50, p65 and CREB2 were immunoprecipitated with an acetyl-lysine antibody. The acetylated proteins (**A**) and the protein expression (**B**) of p50, p65 or CREB2 were analyzed by Western blot. (**C, D**)**,** SW480 cells were pretreated with a p300-selective inhibitor roscovitine (Rosc, 20 μM) (**C**)**,** or transfected with a p300-expressing vector (**D**) for 8 hours, and then treated with UA. At 40 hours after treatment, the cell viability was determined. Empty vector (EV) was used a transfection control. Each bar represents mean ±SD of three experiments. *, *P*<0.05, significant differences between treatment groups and control groups.

The p300-mediated acetylation of transactivators and their binding to COX-2 gene promote play crucial roles in COX-2 expression. We next determined the effect of UA on p300-mediated acetylation of transactivators NF-κB and CREB2 in colon cancer SW480 cells. Treatment with UA at 20 µM in the p300-transfected SW480 cells markedly inhibited the acetylated levels of p50/p65 and CREB-2 proteins (Fig. 6A), whereas the expression of these proteins did not change (Fig. 6B).

To verify the role of UA in regulating the p300 signaling in colon cancer cells, we pretreated with roscovitine, an inhibitor of p300 (Fig. 6C), or transfected with a p300-expressing vector in SW480 cells (Fig. 6D), and the effects of UA on roscovitine or p300-mediated cell proliferation were analyzed. Treatment of cells with roscovitine (Rosc, 20 µM) significantly inhibited cell viability, whereas UA at 20 µM did not significantly change the roscovitine-mediated inhibition (Fig. 6C). By contrast, p300 overexpression significantly reversed the UA-mediated inhibition in SW480 cells by comparison with transfection with an empty vector (EV) (Fig. 6D). These results demonstrate that p300 is an important target of UA and that the UA-induced inhibition of cell proliferation is mediated at least in part through the p300 signaling pathway in colon cancer cells.

### UA Targeted Cytochrome c/caspase Signaling to Induce Cell Apoptosis

**Figure 7:**
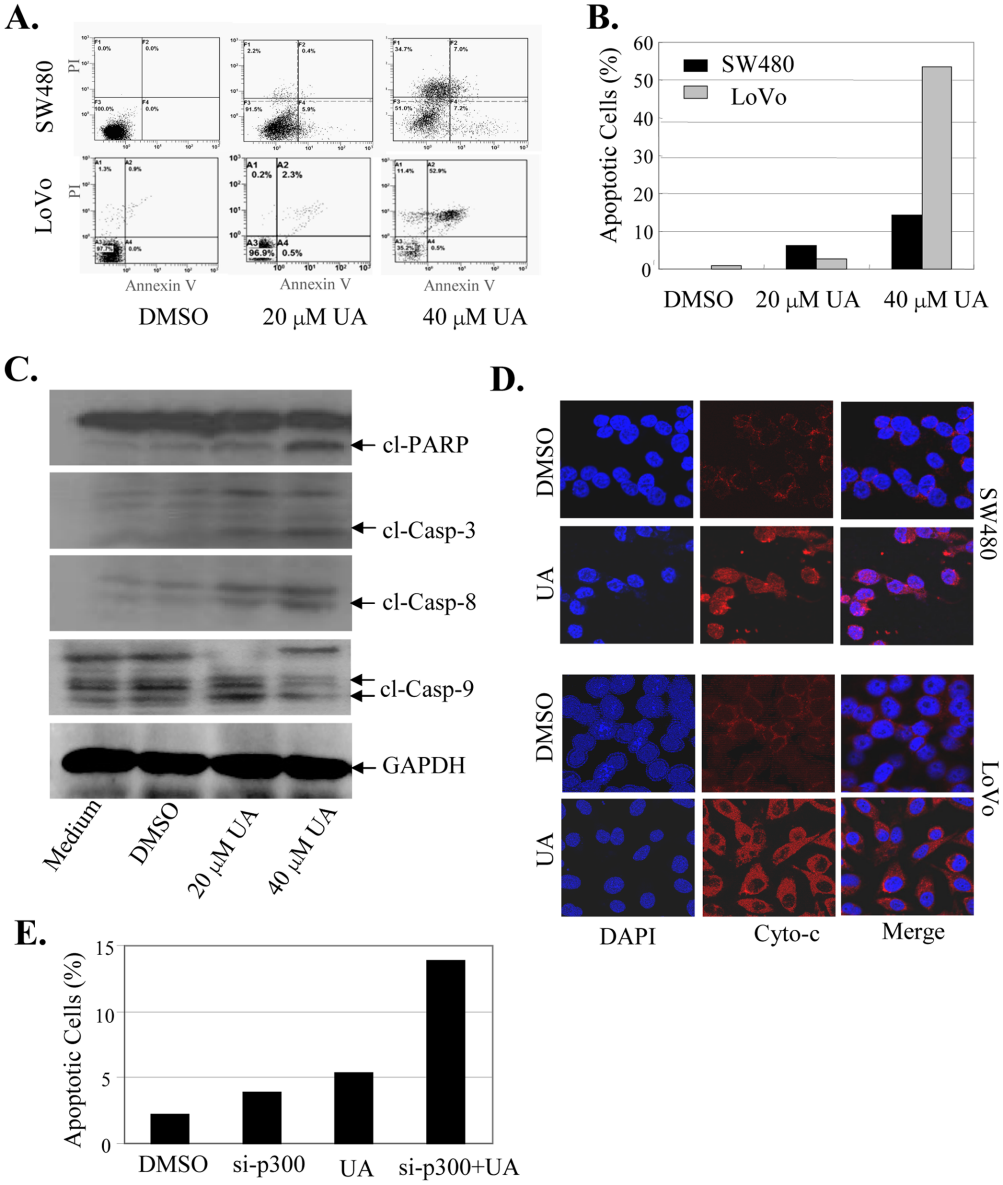
UA activated the cytochroem c/caspase-dependent pathway to induce apoptosis. SW480 and LoVo cells were treated with UA (20 µM and 40 µM). At 48 hours after treatment, the apoptotsis was determined by an AnnexinV-FITC staining-based FACS analysis (**A, B**). The levels of the cleaved caspase-3, caspase-9 and PARP proteins in SW480 cells were analyzed by Western blot (**C**). The release of cytochrome c (Cyto-c) from the inter-mitochondrial space into the cytosol was determined by immunofluorescence imaging analysis (**D**). SW480 cells were transfected with p300 siRNA for 24 hours and followed by the treatment with UA (20 µM). At 48 hours after treatment, the apoptotsis was determined by FACS analysis (**E**). The apoptosis are represented by relative percentages of apoptotic cells versus that in DMSO-treated cells. *, *P*<0.05, significant differences between the UA-treated groups and the control groups.

We also examined the effect of UA on cell apoptosis in colon cancer cells by an Annexin-V staining-based FACS assay at 48 h after treatment. Treatment of cells with UA at 20 µM and 40 µM led to a dose-dependent induction of the positive apoptotic cells (Fig. 7A), leading to a 6.3% to 14.2% induction in SW480 cells and 2.8% to 53.4% induction in LoVo cells (Fig. 7B). Activation of caspases is an important downstream event in apoptotic pathway. We next determined whether apoptosis induced by UA is related to the increased activation of the caspase pathway in SW480 cells. The effects of UA on expression of the cleaved proteins of three key apoptosis-related proteins: PARP, caspase-3 and caspase-9, were detected at 48 hours after treatment by Western blot analysis. As shown in Fig. 7C, treatment with UA at 20 µM and 40 µM resulted in a marked increase of the cleaved PARP, caspase-3 and caspase-9 proteins, suggesting that UA may function as an important and specific mediator to facilitate activation of caspase cascades.

Cytochrome c (Cyto-c) is the upstream molecule of the caspase-dependent apoptosis pathway. We next performed immunofluorescence imaging (IFI) analysis to monitor changes in the subcellular localization of Cyto-c in the UA-treated SW480 and LoVo cells to determine whether UA could trigger Cyto-c release. Treatment with UA (20 µM) effectively promoted the release of Cyto-c from the inter-mitochondrial space into the cytosol (Fig. 7D). These results indicate that UA coordinated Cyto-c release from the inter-mitochondrial membrane space and facilitated the downstream caspase activation in the cytosol.

We also examined the effect of inhibition of p300 on apoptosis induced by UA. The SW480 cells were transfected with a p300 specific siRNA (si-p300) and then treated with UA (20 µM), and the effect of inhibition of p300 on UA-mediated apoptosis were analyzed. The results showed that knockdown of p300 expression by si-p300 markedly enhanced the apoptosis induction mediated by UA (Fig. 7E), confirming the role of p300 signaling in regulating UA-induced apoptosis in colon cancer cells.

## Discussion

In this study, we analyzed the response of human colon cancer cells to UA treatment. UA significantly inhibited cell proliferation, migration and induced apoptosis in a dose-dependent manner. We showed that UA induced cytochrome c release, thereby caused caspase activation and apoptosis. UA also regulated the expression of MMP9 and CDH1 genes and inhibited the phosphorylation of Akt and ERK proteins, leading to an inactivation of the cell migration and proliferation-related signaling pathways. Our study also showed that UA suppressed COX-2 expression and PGE2 production. Furthermore, we showed that the UA-mediated suppression of COX-2 expression and cell proliferation is mediated by simultaneous modulation of the p300, NF-κB, and CREB2 signaling. UA inhibited p300-mediated acetylation of NF-κB and CREB2, and also promoted translocation of the NF-κB p65 and p300 from cell nuclei to cytoplasm. To our knowledge, this is the first report that demonstrated that UA simultaneously targets the multiple signaling pathways to inhibit colon cancer cell proliferation and induce apoptosis.

UA has been intensively studied in the past; mainly as an anti-cancer compound and for its cardiovascular protective properties. The controversy of reports suggests anti-angiogenic and cytotoxic effects of UA on one side and cardiovascular and endothelial protective effects on the other side. Currently, there is insufficient evidence to recommend an ideal human dose. Animal studies suggest benefit use between 0.05–0.2% of the rat diet as UA. Assuming a range of 10–40 mg/kg bodyweight, the benefits in rat studies associated with UA are about equal to a human dose of 1.6–6.4 mg/kg bodyweight (110–440 mg for a 150 lb person).

The side effects of UA from mainly include two aspects: male fertility and DNA damage. Previous study has revealed that UA has the potential of sperm motility inhibition and can serve as a topical vaginal contraceptive [24], [25]. Specifically, it causes breaking of bridges in between cells that are soon to be sperm, and the damaged cells then collect to form symplasts in the seminiferous tubules that are associated with male infertility. UA as an anti-angiogenesis, anti-cancer and locally applied cardiovascular drug may be helpful. However, the DNA damaging activity of UA may also constitute a serious problem. UA was able to induce cell death in endothelial cells when the concentration exceeded 12.5 µM [26].

The expression of COX-2 plays a key role in human cancers. COX-2 expression and PGE2 production have been shown to upregulate the PI3K/Akt and ERK signaling, thereby induced angiogenesis, cell proliferation, migration and invasion [27], [28]. COX-2 inhibition may result in cell growth suppression and apoptosis and induce epithelial-mesenchymal transition [29]–[32]. However, the mechanism by which COX-2 is highly expressed in tumorigenesis is not completely understood. COX-2 transcriptional regulation has been extensively characterized [33]–[35]. P300 has been shown to be essential for COX-2 expression and exerts a global effect on COX-2 promoter chromatin structure to enhance binding of transactivators [20], [21]. P300 is expressed in abundance in cancer cells and p300 overexpression augments COX-2 transcriptional activation [36], [37]. It serves as a transcription coactivator to bridge the promoter-bound transactivators with transcriptional factors in the transcription machinery [20], [21]. P300 HAT acetylates histones thereby opening the chromatin structure and increasing access of enhancer elements to transactivators. P300 HAT is also capable of acetylating transactivators such as NF-κB, thereby enhance transactivator binding [21]. Consistent with the previous reports, our present study also showed that p300 plays a crucial role in regulating NF-κB and CREB2 acetylation and cell proliferation in human colon cancer cells. UA targets p300 signaling to inhibit acetylation of NF-κB and CREB2, thereby inhibit cell proliferation. It has been shown that p300 HAT is regulated by p300 phosphorylation or acetylation [38]. It is possible that UA suppresses p300 phosphorylation, alters p300 HAT conformation and catalytic activity, and inhibits the post translational modification of p300 HAT, thereby blocking the HAT activity in colon cancer cells. Further studies are needed to elucidate the mechanism by which UA inhibits p300 signaling.

Previous studies showed that UA induces apoptosis in human cancers [39]. However, the precise molecular mechanisms and signaling pathways by which UA induces apoptosis remain unclear. Interestingly, our data demonstrate that the induction of apoptosis by UA is mainly through UA-mediated activation of the caspase-dependent apoptotic pathway. Treatment of cells with UA triggered the release of cytochrome c (Cyto-c) to the cytosol, and then stimulated the activation of caspases, thereby induced apoptosis.

We also found that UA targeted the Akt and ERK-dependent signaling pathways to inhibit colon cancer cell proliferation. The phosphorylated Akt and ERK are the mediators of cell proliferation and survival and clinical response to tyrosine kinase inhibitors (TKI), and a reduction in phosphorylated Akt and ERK expression is an important event in sensitizing tumor cells to TKI treatment. UA inhibited the phosphorylation of Akt and ERK proteins, resulting in an inactivation of the cell growth and survival-related Akt/ERK signaling pathway. Our findings have important implications in exploring novel therapeutic strategies for colon cancer.

**Figure 8:**
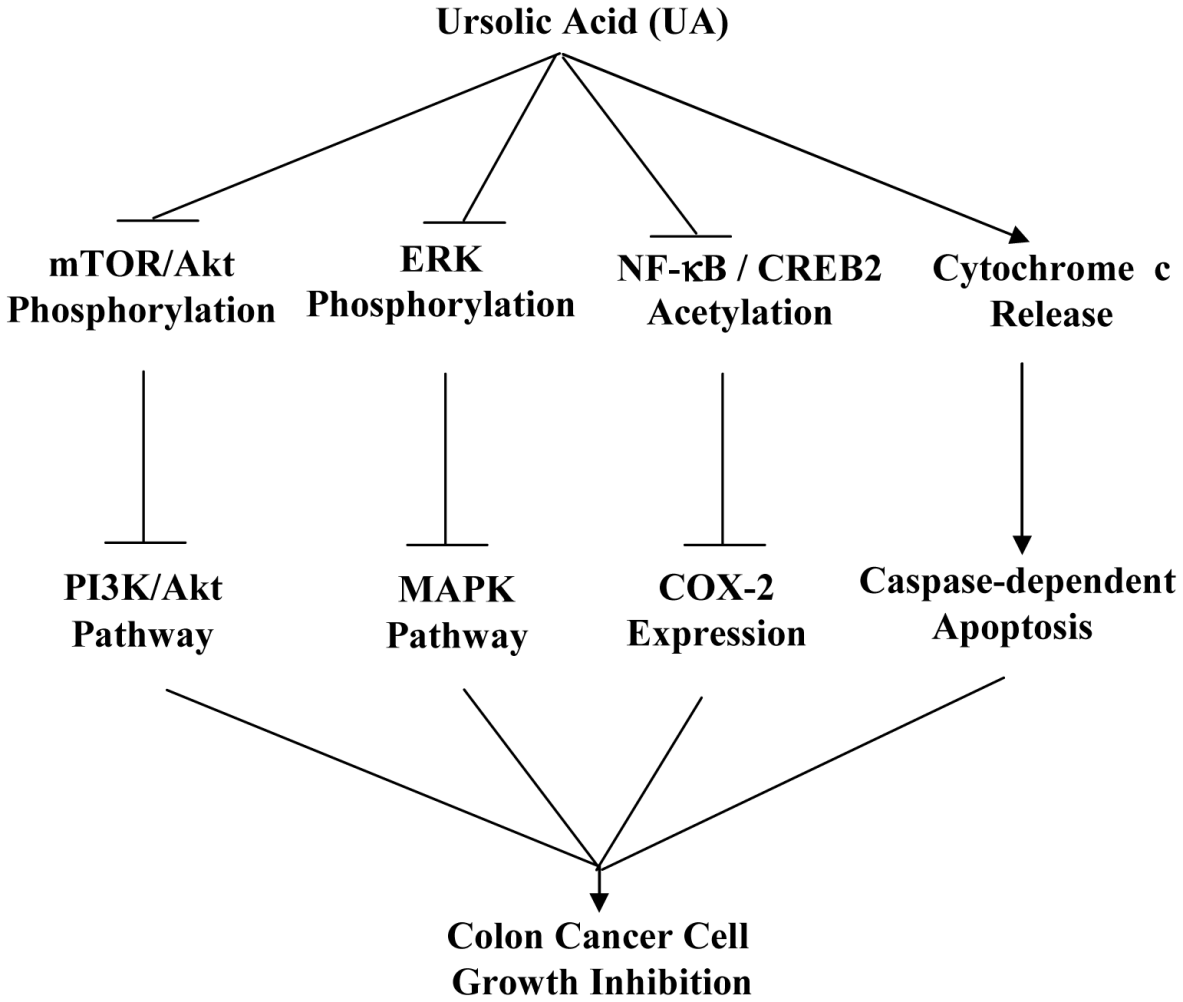
The proposed mechanisms by which UA suppress proliferation and induce apoptosis in colon cancer cells. UA inhibited cell proliferation and migration and induced apoptosis in colon cancer cells by simultaneously modulating the MMP9/CDH1, Akt/ERK, COX-2/PGE2, p300/NF-κB/CREB2, and cytochrome c/caspase-dependent signaling pathways.

In summary, we demonstrated that UA inhibited cell proliferation and migration and induced apoptosis in colon cancer cells by simultaneously modulating the MMP9/CDH1, Akt/ERK, COX-2/PGE2, p300/NF-κB/CREB2, and cytochrome c/caspase-dependent signaling pathways (Fig. 8). Our findings provide new insights into the molecular mechanisms of UA-mediated proliferation inhibition and apoptosis induction and suggest that UA may be a promising agent for the prevention and treatment of human colon cancer.

## Materials and Methods

### Cell Culture and Chemicals

Human colon cancer cell lines (SW480, LoVo) were obtained from the American Type Culture Collection (ATCC, Manassas, VA) and cultured in RPMI 1640 media supplemented with 10% heat-inactivated fetal bovine serum, 100 µg/ml penicillin, and 100 µg/ml streptomycin, and maintained in an incubator with a humidified atmosphere of 95% air and 5% CO_2_ at 37°C. In all experiments, cells with 60% confluence were tested. Ursolic acid (UA), celecoxib and roscovitine were purchased from (Sigma (St. Louis, MO). A 100 mM solution of drug was prepared in dimethyl sulfoxide (DMSO), stored as small aliquots at −20°C and then diluted as needed in cell culture medium.

### **C**ell Viability Assay

Cell viability was determined by MTT assay (Roche Diagnosis, Indianapolis, IN). Briefly, cells plated in 96-well plates (3000 cells/well) were treated with UA at various doses. At 48 h after treatment, cell viability was determined.

### Colonogenic Assay

Cells plated in 6-well plates were treated with UA. After 24 h, cells were washed with PBS and trypsinized. Then cells were seeded at 100 cells/well in 6-well plates, and dispersed evenly by slightly shaking the dishes and incubated at 37°C with 5% of CO2 for 21 days. The medium was discarded and the cells were carefully washed with PBS twice. After being fixed with 4% paraformaldehyde for 15 min, the cells were stained with 0.1% crystal violet for 15 min before washing with tap water and air-drying. The clones with more than 50 cells were counted with an ordinary optical microscope. The clone formation rate was calculated with the following formula: Plate clone formation inhibitory ratio = (number of clones treated with UA/number of cells inoculated)×100%.

### Scratch Assay

Scratch assay was performed to detect cell migration. The cells were grown to full confluency in six-well plates and incubated overnight in starvation medium. Cell monolayers were wounded with a sterile 100 µL pipette tip, washed with starvation medium to remove detached cells from the plates. Cells were treated with the indicated doses of UA in medium and kept in a CO_2_ incubator. After 56 h, medium was replaced with phosphate-buffered saline (PBS) buffer, the wound gap was observed and cells were photographed using an Olympus microscope fitted with digital camera.

### Apoptosis and Cell Cycle Analysis

The UA-treated cells (1×10^5^ cells/ml) were stained with AnnexinV-FITC using an Alexa Fluor® 488 annexin V/Dead Cell Apoptosis Kit (Invitrogen, USA), placed on room temperature for 15 min in the dark, and then analyzed by flow cytometry (Cytomics FC500 Flow Cytometry; Beckman Coulter). Apoptosis was calculated in terms of the AnnexinV-positive in cells. Cell cycle analysis was performed using PI. The transfected cells were harvested with trypsinization, fixed with cold 70% ethanol at 4°C for 12 hours. The staining was performed according to the producer’s manual. Raw data were analyzed using CXP Software and Multicycle for Windows (Beckman Coulter).

### Western Blot Analysis

Cell lysates were separated by electrophoresis in a 10% sodium dodecyl sulphate-polyacrylamide gradient minigel (SDS-PAGE) and electrophoretically transferred to a PVDF membrane. Western blots were probed with specific antibodies against phospho-PI3K, PI3K, phospho-Akt, Akt, phospho-mTOR, mTOR, phospho-PTEN, PTEN, phospho-JNK, JNK, phospho-ERK1/2, ERK1/2 (Cell Signaling Technology), GAPDH, PARP, Caspase-3/9, COX-2, p300, NF-κB p50 and p65, CREB-2 and cytochrome c (Santa Cruz Biotech, Santa Cruz, CA). The protein bands were detected by enhanced chemiluminescence (Pierce).

### RNA Purification and Real-time PCR Analysis

Total RNA was isolated using TRIZOL reagent (Invitrogen, Carlsbad, CA,USA), and DNase I-treated RNA (2µg) was used for first-strand cDNA synthesis. Quantitative real-time PCR was done in an ABI PRISM 7900 HT sequence detection system with the preset PCR program, using SYBR® Premix Ex TaqTM II (TAKARA code DERR081A). The primer sequences used in real-time PCR were: MMP9, 5′- GGGACGGCAATGCTGAT-3′ and 5′-CGCCACGAGGAACAAACT -3′; CDH1-5′-CGCATTGCCACATACACTCT-3′ and 5′CGGGCTTGTTGTCATTCTG -3′; COX-2 5′-TCACAGGCTTCCATTGACCAG-3′ and 5′-CCGAGGCTTTTCTACCAGA-3′, GAPDH,5′- GCACCGTCAAGGCTGAGAAC -3′ and 5′- TGGTGAAGACGCCAGTGGA -3′. The reaction was done in a total volume of 50 µl, with 40 ng of cDNA and 2µl of each primer in 1 * SYBR® Premix. Quantitative data was analyzed by the ratio between the value of MMP9, CDH1, COX-2 and GAPDH. Conditions for all PCRs were optimized for a 50-µl reaction using the following 40 cycle program: step1∶95°C for 30 sec repeat 1 cycle; step2∶95°C for 5 sec, and 60°C for 30 sec repeat 40 cycles. All samples were amplified simultaneously in triplicate in one assay-run. GAPDH was included in each reaction as an internal standard and relative quantitative gene expression was calculated.

### Reverse Transcription-polymerase Chain Reaction (RT-PCR)

Total cellular RNA was isolated using the Trizol® reagent, according to the manufacturer’s protocol (Invitrogen Corporation, Carlsbad, CA). Total RNA (2.5 µg) was reverse-transcribed by using the Superscript™-III kit (Invitrogen, Carlsbad, CA). PCR analysis was performed on aliquots of the cDNA preparations to detect gene expression. PCR conditions were 4 min at 94°C followed by 35 cycles (25 for GAPDH): 30 seconds at 94°C, 30 seconds at 60°C, and 1 min at 72°C,

### Transfection

The transfection of p300-overexpressing vector or p300 siRNA (Santa Cruz Biotechnology, Santa Cruz, CA) was performed by Lipofectamine 2000 reagent according to the manufacturer’s protocol (Invitrogen, Carlsbad, CA).

### Immunofluorescence and Confocal Imaging

Cells grown on chamber slides were washed in phosphate-buffered saline and fixed for 15 min at room temperature with 4% paraformaldehyde. The samples were pretreated with 10% bovine serum albumin (BSA) in PBS for 30 min. Antibodies against p300, p50, p65 or cytochrome c in the blocking solution were added to the sample and incubated for overnight at 4°C. Nonimmune rabbit IgG and mouse IgG were included as controls. Following five 5-min washes with PBS, fluorescein isothiocyanate- and rhodamineconjugated secondary antibodies were added in blocking solutions and incubated for 1 hour. After five additional 5-min washes, samples were examined with a Zeiss LSM700 confocal microscope, and images were processed with Zen 2010 software. More than 100 cells were inspected per experiment, and cells with typical morphology were presented.

### Acetylation Analysis

Cells were treated with UA for 24 h. The nuclear extracts prepared from the treated cells were immunoprecipitated with a specific antibodys against acetyl-lysine and the immunoprecipitate was pulled down with protein A/G agarose beads (Santa Cruz Biotechnology, Santa Cruz, CA). After extensive washing, proteins were separated in a 10% SDS-PAGE system and the acetylated p50, p65 or CREB2 was detected with a monoclonal antibody against p50, p65 or CREB2 (Cell Signaling Tech., Beverly, MA).

### Statistical Analysis

Analysis of variance and Student’s *t* test were used to compare the values of the test and control samples. *P<0.05* was considered to a statistically significant difference. SPSS6.0 software was used for all statistical analysis. The significance was evaluated by the paired *t* test. All the experiments were done three times, and mean values and standard deviation were calculated.
